# Investigation of maternal breastfeeding guarantee policy needs and influencing factors: a cross-sectional study in China

**DOI:** 10.3389/frhs.2024.1348888

**Published:** 2024-03-07

**Authors:** Junying Li, Lan Zhang, Nafei Guo, Ying Liu, Hui Jiang

**Affiliations:** ^1^ Nursing Department, Shanghai First Maternity and Infant Hospital, Tongji University School of Medicine, Shanghai, China; ^2^ Outpatient Office, Shanghai First Maternity and Infant Hospital, Tongji University School of Medicine, Shanghai, China

**Keywords:** breastfeeding, policy protection, maternal and infant rights, policy needs, China background

## Abstract

**Background:**

The promotion of breastfeeding is an important strategy to prevent neonatal death and improve maternal and infant health. But Chinese efforts to improve breastfeeding practices have not been particularly effective. There is still a long way to go to achieve the national health development goals. We aimed to explore the maternal demand for breastfeeding guarantee policy in China and to determine the impact of a range of socio-demographic and neonatal-related variables on breastfeeding guarantee policy demand.

**Methods:**

The study was carried out in the Obstetrics and Gynecology hospital of Shanghai, one of China's earliest provincial and municipal maternal and child health care institutions. From June to November 2021, 1,292 women were recruited for the cross-sectional study in child health clinic. We collected relevant socio- demographic and neonatal-related data. Maternal breastfeeding needs were measured through a self-designed questionnaire on breastfeeding guarantee policy demands of mothers.

**Results:**

The mean score of breastfeeding guarantee policy demand was 4.42 ± 0.51. There were statistically significant differences in the effects of maternal age, education level, family income per capita (Yuan), medical payment type, baby age, work status, and current feeding methods on the demand for breastfeeding guarantee policies (*P* < 0.05). Multiple linear regression analyses showed that higher education level (*B* = 4.437, *P* < 0.001), baby age (*B* = 2.150, *P* = 0.002), and current feeding methods (*B* = 2.754, *P* = 0.005) were significantly associated with a higher demand for a breastfeeding guarantee policy, the effect of medical payment type is the most influencing factor (*B* = −7.369, *P* < 0.001).

**Conclusions:**

The maternal needs for breastfeeding guarantee policy are multi-faceted and urgent. In the process of improving and implementing policies, the government and relevant departments should take into account the actual needs of women who have different education levels, baby ages, family economics, and feeding methods.

## 1 Background

Breastfeeding is the gold standard for neonatal and infant feeding, and breastfeeding is the best form of nutritional supply early on in human life (1). The World Health Organization (WHO) and the United Nations International Children's Emergency Fund (UNICEF) recommend that children start breastfeeding within one hour of birth, and exclusively breastfeed for six months, and suggest sustainable breastfeeding until the child is two years old or older (2). Breastfeeding plays an important role in ensuring the health and survival of mothers and babies. However, global rates of breastfeeding and exclusive breastfeeding remain low. According to the UNICEF global database, as of 2020, only about 48% of infants worldwide are breastfed within one hour of birth, and the average rate of exclusive breastfeeding for infants aged 0–5 months is 44% (1). The rate of exclusive breastfeeding within the first six months is 28.6% in Mexico, 18.8%−55.0% in the United States and 29.0% in Australia, but only 21% in China (3–5).

In order to improve the national breastfeeding behavior in China, all parties have made a series of efforts, such as medical institutions providing professional breastfeeding knowledge (e.g., lack of lactation, engorgement), skills (e.g., breastfeeding position, latch) and personalized care (e.g., skin to skin, room-in, milk expression) (6, 7), and the community providing support through telephone follow-up, peer education, and family counseling (8, 9), as well as other support through mobile medical and psychological interventions (10, 11). The results were mixed. However, the research on maternal and infant rights and breastfeeding-related safeguarding policies is not widely used.

Breastfeeding is the biological natural ability of almost all mothers, but in real social life, breastfeeding behavior is often restricted by a variety of influencing factors, among which policy factors often play a guiding role (12). Research has shown that health worker counselling and guidance are the relatively most visible policy measures and guidelines affecting infant feeding (13).

Increased maternity leave is strongly associated with long-time breastfeeding (14), and ensuring no less than six months of paid maternity leave can increase breastfeeding rates by 8.9% (15). In addition, the continued breastfeeding rate among women after returning to work will also greatly increase when maternity leave is paid or child care and support are provided (16). Moreover, the workplace providing private breastfeeding spaces can also effectively reduce the breastfeeding barriers of working women (17). In Alabama, United States, the local Department of Public Health has created a number of “baby cafes” with the support of the Breastfeeding Council to provide a good social space for breastfeeding (18). The Brazilian government has initiated a breastfeeding promotion plan to publicize the importance of breastfeeding through the mass media, making the public aware of the urgency of increasing breastfeeding rates and controlling the promotion and marketing of breastmilk substitutes, such as formula milk. It has also achieved impressive results in promoting breastfeeding behavior (19).

In China, the government has gradually attached the importance of promoting and developing of breastfeeding since the 1980s. In 1990, the Ministry of Health clearly proposed to carry out extensive breastfeeding education and scientific research, and established 5.20 every year as the National breastfeeding promotion day (20). Since 1992, in response to the WHO's call, China has begun to promote the construction of baby-friendly hospitals and then actively formulated and implemented the “Measures for the Administration of Marketing of Breastmilk Substitutes” (1995) and the “Measures for the Implementation of the Maternal and Infant Health Law”. In the second revision of the “Maternal and Child Health Law of the People's Republic of China” in 2017, the maternal and child health care part includes: maternal and child health guidance; pregnant women care (provide pregnant women with health/nutrition/psychological consultation or guidance as well as regular prenatal examination); fetal health care and newborn health care (21).

In June 2021, the State Council officially issued a decision on optimizing childbirth policies to promote long-term balanced population development, and the three-child policy was proposed. And in the “Outline for Women's Development in China (2021–2030)” and “Outline on the Development of Chinese Children (2021–2030)”, they are proposed that employers should strengthen the protection of breastfeeding support for female employees, such as strengthening the protection of breastfeeding, providing time and space for breastfeeding, and ensuring maternity leave according to law; in terms of children's health care, it is proposed to encourage and promote medical institutions to set up “pregnant women schools”, parent classes and other popular science education activities; emphasizing the management of baby-friendly hospitals, expanding the number and coverage of maternal and infant facilities, and striving to achieve the target of 50% exclusive breastfeeding of infants within 6 months (22). In November of the same year, the state released for the first time a plan aimed only at promoting breastfeeding actions-“Notice on Printing and Distributing the Breastfeeding Promotion Action Plan (2021–2025)”, which started from building a social breastfeeding promotion network; focusing on breastfeeding consultation and guidance during pregnancy, childbirth, and breastfeeding; exploring the unique advantages of traditional Chinese medicine in breastfeeding promotion (23).

In addition, in order to better protect the rights of women and children, according to the “Special Rules on the Labor Protection of Female Employees”, female employees can enjoy 98 days of paid maternity leave, including prenatal leave 15 days. And each local government has made different local provision on female workers maternity leave and related treatment though the local “population and family planning management regulations”, with an average maternity leave of 158 days and 15 days for male paternity leave (24). The promulgation and implementation of these policies aroused widespread repercussions in society at that time and played a significant role in increasing the breastfeeding rate in China.

Although the Chinese government has made decades of efforts to improve breastfeeding practices, the current breastfeeding guarantee policies do not match the needs of the masses, and the relevant implementation is not yet in place. In the real social environment, it is difficult to achieve qualitative changes to greatly increase the exclusive breastfeeding rate and achieve the goal of “Healthy China 2030”, which, in the absence of breastfeeding-related hardware security facilities, means creating documents and leading efficient publicity. Therefore, in view of the current situation of breastfeeding in China, it is of practical significance to carry out relevant research from the perspective of a breastfeeding security policy.

Therefore, this study focuses on the strong driving effect of policy factors on breastfeeding, also, explores the needs of mothers for a breastfeeding guarantee policy and examined other relevant factors through a literature review and preliminary qualitative research. The study further proposes corresponding countermeasures and suggestions according to the voices of maternity in order to provide a reference for breastfeeding-related clinical treatment, as well as nursing and social intervention strategies. Also, the results are useful in the promotion of maternal and infant health and in efforts aimed at increasing the breastfeeding rate.

## 2 Methods

### 2.1 Design

This was a quantitative analytic study using a cross-sectional survey.

### 2.2 Setting

The study was carried out in Shanghai, one of China's largest cities and economic centers. The subjects were recruited from one of the Third-A-Class obstetrics and gynecology hospitals, which is one of the earliest provincial and municipal maternal and child health care institutions in China. It has more than 800 beds and a maximum annual delivery capacity of 30,000 (Shanghai First Maternity and Infant Hospital, 2020, and is known as the cradle of the new life in Shanghai). The convenience sampling method was used to find potentially suitable participants. The potential subjects had the right to refuse or withdraw from their participation in the study at any time. Importantly, they did not face any reprisal for doing so.

### 2.3 Ethical considerations

The study received ethical approval from the hospital's Institutional Review Board (KS21217). After informed consent was obtained from the subjects, women filled in an anonymous electronic questionnaire using their smartphone under the guidance of researchers. And the research purpose, significance, potential impact, and confidentiality of personal information were communicated to subjects in the participant information interface. All methods were performed in accordance with the relevant guidelines and regulations including the Declaration of Helsinki.

### 2.4 Recruitment and sampling

From June to November 2021, maternity women were recruited for the study during routine clinical physical examinations of neonates in the postpartum period (usually 0–24 months postpartum). The convenience sampling method was used to find potentially suitable participants. The inclusion criteria for the sample were as follows: women were at least 20 years old; full-term; live birth (gestation ≥37 weeks); the babies were aged 0–24 months; and the women voluntarily agreed to participate in the study. The exclusion criteria were women who had a history of mental illness and breastfeeding contraindications; used drugs that affect lactation; or their babies had congenital malformations; or refused to participate to the study.

We calculated the sample size according to the sample size calculation formula of the descriptive study design cross-sectional survey (25), taking *α* = 0.05, the allowable error *δ* = 3%. The exclusive breastfeeding rate within 6 months was 21% in China, according to the literature review (*π* = 21%). The sample size was calculated to be 710 cases. Taking into account the loss and failure of samples, the sample size was increased by 15%, and the final sample size was 820 cases.

$$n=\frac{\mu_{\alpha /2}^{2}\pi (1-\pi )}{\delta^{2}}$$

### 2.5 Study design and the survey

#### 2.5.1 Research instrument

This study is guided by the theory of policy tools and based on the classification introduced by Rothwell and Zegveld, which divides policy tools into three types: supply type, demand type, and environmental type (26). The first two types of policy tools directly promote the development of breastfeeding, and environmental policy tools have an indirect guiding role. It simplifies the complex policy system, not deliberately emphasizing the coercion of the government but rather highlighting the leading environmental role of relevant departments in promoting the development of a certain field.

Based on the policy tool theory, our research group created the questionnaire to assess the need of policy support, and named it the “Breastfeeding Guarantee Policy Demands of Mothers questionnaire”. According to Chinese national conditions and the medical environment, the initial item pool of the questionnaire was determined based on a literature review and previous qualitative research, and adapted by senior obstetricians, neonatologists, international lactation consultants, and senior clinical nurses. Finally, all of the above agreed on the final version of the questionnaire in terms of its content and face validity. The Cronbach' s *α* was 0.968 overall.

#### 2.5.2 The socio- demographic characteristics

When women took their babies to the child health care clinic for evaluation, the researchers collected the following mothers' and babies' data: age, education, work situation, family economy, baby age, current feeding method (exclusive breastfeeding, mixed feeding, formula feeding), maternity leave time, paternity leave time.

#### 2.5.3 Breastfeeding guarantee policy demands of mothers questionnaire

The questionnaire includes three dimensions: supply-type policy need, demand-type policy need, and environmental policy need, involving medical institution support, the construction of baby care rooms, workplace support, government support, maternity leave support, cultural breastfeeding publicity, and management of breast milk substitutes. There were seven aspects of such requirements and a total of 45 items. We used a 5-point Likert scale (1 = “no need at all”, 2 = “not a very strong need”, 3 = “general need”, 4 = “moderate need”, 5 = “strong need”), with a total score of 0–225. The total score was obtained by summing the item scores, the higher the score, the higher the demand of the mother for breastfeeding protection policies, and the higher the possibility of further promotion of breastfeeding. The Cronbach' s *α* of each dimension was between 0.827–0.965, and the average content validity of the questionnaire was 0.98, revealing excellent internal reliability and validity.

### 2.6 Data analysis

Data were entered with Excel 2016 and analyzed with IBM SPSS Statistics 21.0. The general data of the research subjects are described by frequency, percentage, means (*M*) and standard deviation (*SD*). The maternal demand for breastfeeding guarantee policies is presented as the *M* and *SD*. The normality test of the data shows that the score distribution of maternal demand for breastfeeding guarantee policies is non-normal. Thus, this paper used the statistical analysis method of a non-parametric test to compare the difference of the maternal demand for breastfeeding guarantee policies. We used a multiple linear regression analysis to explore the relationship between influencing factors and demand. All statistics were performed using a two-sided test, the test level was *α* = 0.05, and the statistical significance was set a *P*-value <0.05.

## 3 Result

### 3.1 Sample characteristics

The characteristics of the participants are shown in Table 1. We contacted 1,503 participants, some of whom chose to drop out due to other reasons, such as time or health constraints, missing questionnaires, or a filing time that was too short. Finally, we collected 1,292 cases from whom questionnaires were recovered, with an effective recovery rate of 85.96%. The average maternal age was 30.76 ± 3.54. In total, 68.8% of participants had a bachelor's degree or higher, 78.8% were primiparas, 17.5% of babies were ≥4 months old, and the rate of those who exclusively breastfed was 45.9%. Also, 64.65% of the maternity were working (including freelancers), and most of them were still taking maternity leave. Full-time mothers accounted for 15.7%, but 16.1% of the participants said they didn't know whether they had maternity leave, 31.0% of the maternity didn't know whether they had breastfeeding leave, 11.4% said they did not have breastfeeding leave, 22.1% did not know whether their spouses had paternity leave, and 9.7% said that their spouses did not have paternity leave.

**Table 1:** Comparison of maternal demand for breastfeeding guarantee policies among different socio- demographic characteristics (*n* = 1,292).

Variables	*n*	%	*Z*/*χ*^2^	*P*
Age				
<25 years	46	3.6	17.818^b^	0.000**
25–30 years	423	32.7		
30–35 years	640	49.5		
≥35 years	183	14.2		
Education level				
Junior middle school or below	48	3.7	71.252^b^	0.000**
High school or technical secondary school	93	7.2		
College	262	20.3		
University	631	48.8		
Master or above	258	20.0		
Family income per capita (Yuan)				
<5,000	154	11.9	12.011^b^	0.017*
5,000–10,000	516	39.9		
10,000–150,000	324	25.1		
15,000–20,000	147	11.4		
≥20,000	151	11.7		
Household registration location				
Shanghai	406	31.4	169,386.000^a^	0.092
Non-Shanghai	886	68.6		
Type of medical payment				
Medical insurance	75	22.52	64,107.000^a^	0.000**
Own expense	138	41.44		
Parity				
One child	1,018	78.8	135,542.000^a^	0.474
Second child or above	274	21.2		
Mode of delivery				
Natural delivery	747	57.8	198,861.500^a^	0.478
Cesarean section	545	42.2		
Baby gender				
Boy	678	52.1	216,023.500^a^	0.250
Girl	623	47.9		
Baby age				
<42 days	134	10.4	15.040^b^	0.005**
42 days–4 months	931	72.1		
4–6 months	60	4.6		
6–12 months	111	8.6		
≥12 months	56	4.3		
Work status				
Employed (including freelance) and currently returning to work	254	19.7	15.078^b^	0.001**
Employed (including freelance) but currently on maternity leave	835	64.6		
None (including unemployed or stay-at-home mothers)	203	15.7		
Current feeding method				
Exclusive breastfeeding	593	45.9	6.657^b^	0.037*
Mixed feeding	677	44.7		
Formula feeding	122	9.4		
30–35 years	640	49.5		
≥35 years	183	14.2		

^a^ Mann-Whitney *U* analysis.

^b^ Kruskal-Wallis analysis.

* *P* < 0.05.

** *P* < 0.01.

The mean maternity leave was 130.31 ± 13.79 days, with a median of 128 days (four months); the actual breastfeeding leave was 1.6 ± 0.60 h per day, with a median of one hour per day, the expected duration of breastfeeding leave was 2.26 ± 0.94 per day, and the median time was two hours per day; the average length of paternity leave currently enjoyed by their spouses was 12.44 ± 9.03 days, with a median of ten days, and a median expected paternity leave duration of 30 days.

Table 1 shows the influencing factors of the maternal demand for a breastfeeding guarantee policy among different socio- demographic groups. There were seven variables that had an impact on breastfeeding guarantee policy scores, namely age, education, family income per capita (Yuan), type of medical payment, neonatal age in months, work status, and current feeding methods (*P* < 0.05, Table 1).

### 3.2 Maternal demand score for breastfeeding guarantee policy

Table 2 presents the scores of the maternal demand for a breastfeeding guarantee policy. The total score of all items was 198.70 ± 22.97, and the average score was 4.42 ± 0.51 between having a need and a strong need. The supply-type policy need had the highest score; the average score was 4.53 ± 0.51 and the lowest score was found for the environmental policy need, with an average score of 4.24 ± 0.58. In each sub-dimension score, the highest score was found for the demand for the Baby care rooms support (4.7 ± 0.53), followed by the demand for maternity leave support (4.59 ± 0.66), while the lowest score was found for the demand for breastmilk substitute management (4.09 ± 0.80), which showed a significant difference. Table 3 presents the five items with the highest and lowest scores in the questionnaire.

**Table 2:** The score of maternal demand for breastfeeding guarantee policy (*n* = 1,292).

Dimension	Total score		Average score	
	*`* $\bar{X}$	SD	$\bar{X}$	SD
Supply-type policy need	108.82	12.31	4.53	0.51
Medical institution support	62.46	8.03	4.46	0.57
Baby care rooms support	28.34	3.2	4.72	0.53
Workplace support	18.02	2.51	4.51	0.63
Demand-type policy need	30.49	4.61	4.36	0.66
Government support	30.49	4.61	4.36	0.66
Environmental policy need	59.39	8.14	4.24	0.58
Maternity leave support	18.38	2.63	4.59	0.66
Breastfeeding cultural publicity	24.65	4.52	4.11	0.75
Management of breast-milk substitutes	16.37	3.21	4.09	0.80

**Table 3:** The highest and lowest scores for each dimension of breastfeeding guarantee policy demands ($\bar{x}\;\;\pm \;s$).

Dimension	Sub-dimension	Item score			
		The highest item	Score	The lowest item	Score
Supply-type policy need	Medical institution support	Guide breastfeeding knowledge and skills after returning to the ward (such as distributing video materials)	4.60 ± 0.62	Provide postpartum breastfeeding guidance services in the community after discharge	4.36 ± 0.73
	Baby care rooms support	Build infant room in large public places (train stations/airports/shopping malls)	4.78 ± 0.54	The location of the lactation room in the workplace is appropriate	4.63 ± 0.67
	Workplace support	Flexible work arrangements (such as reducing field work, telecommuting from home)	4.56 ± 0.65	Unit leaders and colleagues support breastfeeding	4.38 ± 0.79
Demand-type policy need	Government support	Financial support from the state to promote breastfeeding (such as breastfeeding allowances)	4.45 ± 0.72	Build and develop breast milk banks	4.19 ± 0.88
Environmental policy need	Maternity leave support	Extended paternity leave	4.62 ± 0.71	Extended maternity leave	4.58 ± 0.77
	Breastfeeding cultural publicity	provide the location distribution information of baby-friendly hospital and infant room(such as designing a special query APP)	4.32 ± 0.90	Health administrative departments promote breastfeeding through mass media such as television, websites, WeChat, and Weibo	3.87 ± 0.95
	Management of breast-milk substitutes	Strictly control the publicity and marketing ways of breast milk substitutes (such as gifts/lectures/promotions/using the slogan “Imitating breast milk”)	4.25 ± 0.82	sale of breast-milk substitutes for babies under six months in pharmacies	3.99 ± 1.01

### 3.3 Factors for maternal demand score for breastfeeding guarantee policy

For a better interpretation of the multiple linear regression analysis, the independent variable assignment method is shown in Table 4. Table 5 shows that a higher maternal demand for breastfeeding policy scores was significantly associated with education (*B* = 4.437, *P* < 0.001), neonatal age in months (*B* = 2.150, *P* = 0.002), and current feeding methods (*B* = 2.754, *P* = 0.005). The type of medical payment is the most influencing factor to the higher demand for breastfeeding guarantee policy (*B* = −7.369, *P* < 0.001).

**Table 4:** Multiple linear regression analysis of independent variable assignments on maternal demand for breastfeeding guarantee policy.

Variable number	Variable name	Independent variable assignments
Y	Overall average	Measured value
X1	Age	1 = <25 ages, 2 = 25–30 ages, 3 = 30–35ages, 4 = ≥35ages
X2	Education	1 = Junior middle school or below, 2 = High school or technical secondary school, 3 = College, 4 = University, 5 = Master or above
X3	Family income per capita (Yuan)	1 = <5,000, 2 = 5,000–1,0000, 3 = 10,000–15,000, 4 = 15,000–20,000, 5 ≥ 20,000
X4	Medical payment type	1 = Medical insurance, 2 = Own expense
X5	Work status	1 = Employed (including freelance) and currently returning to work, 2 = Employed (including freelance) but currently on maternity leave, 3 = None (including unemployed or stay-at-home mothers)
X6	Baby age	1 = <42 days, 2 = 42 days–4 months, 3 = 4–6 months, 4 = 6–12 months, 5 = ≥12 months
X7	Current feeding method	1 = Formula feeding, 2 = Mixed feeding, 3 = Exclusive breastfeeding

**Table 5:** Multiple linear regression analysis of factors associated with maternal demand for breastfeeding guarantee policy.

Variables	Unstandardized coefficients		Standardized coefficients	*t*	*P*	95.0% confidence interval for B		Tolerance	VIF
	B	Std. error				Lower bound	Upper bound		
(Constant)	216.639	3.052		70.977	0.000**	210.651	222.626		
Education	4.437	0.687	0.189	6.461	0.000**	3.225	5.784	0.836	1.196
Type of medical payment	−7.369	2.061	−0.103	−3.575	0.000**	−11.412	3.325	0.856	1.168
Baby age	2.150	0.700	0.085	3.070	0.002**	0.776	3.523	0.935	1.069
Current feeding method	2.754	0.969	0.078	2.842	0.005**	0.853	4.655	0.957	1.045

** *P* < 0.01.

## 4 Discussion

### 4.1 High overall demand score

The implementation and development of breastfeeding guarantee policies are beneficial in promoting the growth and development of infants and improving immunity (27). In May 2021, after China's “Three-child Policy” was proposed, the “Decision of the Central Committee of the Communist Party of China and the State Council on Optimizing the Birth Policy to Promote Long-term Balanced Population Development” (hereafter referred to as the “Decision”) clearly mentioned the promotion of prenatal and postnatal care and the implementation of breastfeeding promotion (28). Maternal mothers have certain needs for breastfeeding knowledge, skills, duration of breastfeeding, and breastfeeding support at the social level (29, 30) similar to the results of this study. Breastfeeding policy could improve maternal women breastfeeding competency, which directly or indirectly affects breastfeeding behaviors. The Vietnamese government's integration of high-quality interpersonal counseling services into health system services has greatly improved the breastfeeding rate in the country (31). Alabama Breastfeeding Commission (ABC) developed breastfeeding services in the community. This service was developed in the form of lactation group during the perinatal period, aiming to solving feeding problems at the grassroots level and form a support network. It did a great job of improving breastfeeding practices in Alabama (32).

### 4.2 Urgent need for construction of baby care room

It is undeniable that constructing baby care rooms in public places is one of the important symbols of social civilization. Following the “Two-child Policy”, China has implemented the “Three-child Policy”, which will undoubtedly bring increased fertility, and the society's demand for childbearing support will also increase day by day. It has been documented that having a well-equipped nursing space and adequate nursing time increases the likelihood of breastfeeding by 2.3 times for working women (33). The results of our study showed that women have the highest scores for the item of “construct a baby care room in large public places (such as train stations/airports/shopping malls)” and “protect the privacy in breastfeeding spaces”, which indicated that women urgently need sufficient and quality-assured baby care rooms to support their breastfeeding. The findings of this study are similar to those of Ahmad et al., they found that women desired to continue breastfeeding after working if employers could provide facilities and flexible hours for expressing milk during working hours (34). The addition of the “Baby Cafe” in Alabama provides a good social space for the implementation and communication of breastfeeding (32). The Australian government's welcoming breastfeeding programme in public spaces has had a positive impact and increased public awareness of breastfeeding (35). However, compared with the mature and user-friendly standards for the equipment of baby care rooms in foreign countries (36), there are obvious deficiencies in the planning, equipment, and management of public facilities, such as baby care rooms in China.

### 4.3 Valuing the support needs of medical institutions

The postnatal breastfeeding experience during hospitalization is a key factor influencing lactation and the continuation of breastfeeding (37). Our study showed that breastfeeding is a behavior that requires study and support, especially for primiparas. Medical professionals are their most trusted group, and women are eager to acquire breastfeeding-related knowledge and skills from them. Nieuwoudt pointed out that consultation and guidance from health worker are the most obvious policies and guideline for infant feeding (13). Some studies have found that strengthening counseling and attention to maternal psychology at hospital level was important to promoting breastfeeding. It can help to learn their real need, also, avoid forced or mechanical feeding (38). Also, maternity trust that medical staff will pay close attention to their own emotional and psychological changes, rather than just paying attention to the newborn.

However, in the reality of busy clinical work, medical staff often neglect this. Therefore, medical staff should focus on communicating with maternity to understand their real needs using methods to encourage them to continue breastfeeding, such as sharing knowledge and imparting experience and skills (39). This should be done in an effort to improve the quality of nursing care for clinical breastfeeding.

Coincidentally, the study also found that most women had a low demand for prenatal education and community support for breastfeeding. Studies have also shown that maternal lack the prospective awareness of breastfeeding, mainly because of their lack of insight into breastfeeding (29). They are unprepared for the potential difficulties of breastfeeding, and cannot imagine the gap between breastfeeding ideal and reality.

Prenatal education and community-based health care services in China are still in the exploratory stage. Most women have insufficient awareness of prenatal education and community support, and some even think that it is unnecessary. The qualitative interview previously conducted also verified this result (40). However, national and international studies have proven that prenatal education has a significant effect on improving women's breastfeeding willingness, postpartum lactation initiation, and breastfeeding rate (41, 42). Developing community support for breastfeeding cannot only effectively relieve the pressure of medical treatment and follow-up in major hospitals but also systematically manage continuous maternal care services and save medical resources, thereby effectively protecting the rights and interests of mothers and babies (43, 44). Therefore, how to balance maternal breastfeeding demands and practical problems in work, and improve the ways to support education, are one of the directions of future research.

### 4.4 Emphasizing government support needs, but low demand for content beyond traditional concepts

Government support for breastfeeding significantly improves the initiation and continuation of breastfeeding. Study has shown that government financial support has had an important impact on the development of baby-friendly hospital initiatives and the promotion of breastfeeding (45). In Mexico, government leaders emphasized the need to increase the budget for the Integrated Nutritional Nursing Strategy health work, develop breastfeeding funds, and use maternity allowances (46). However, in China, financial support has not followed up and incentives have not been developed, while the government has chosen to make breastfeeding a priority for development, as this study also confirms.

Our study further finds the interesting result that the item of “build and develop breast milk banks” has the lowest score. Maybe other women's milk is often regarded as “unclean”, “carrying genetic disease”, or “dirty” in traditional Chinese concepts. It is difficult for most mothers to accept donated breast milk from the breast milk bank, both psychologically and physically. In 1980, both the WHO and UNICEF had proposed that the first choice for a baby's food source is donor milk when the mother cannot breastfeed herself (47). However, the first breast milk bank in China was built in 2013, and it is still in its infancy (48). It is undeniable that the development of breast milk banks plays a very important role in promoting breastfeeding, especially in improving breastfeeding and the prognosis of premature infants. Therefore, future research should vigorously develop and promote the use of breast milk banks on an evidence-based basis. This may lead more Chinese women to accept and be willing to use donor milk and ensure the proper nutrition of infants and young children.

### 4.5 Strong need for childbirth leave protection

In this study, mothers had a higher demand for extended maternity leave, breastfeeding leave, and paternity leave. For nearly half a century, growing academic research has focused on the important role of fathers in fertility and infant care (49, 50). The research of Petterson et al., show that both genders expressed interest in expanded paid maternity and paternity leave (51). Some organizations' leaders in the field have suggested that increasing paid childbirth leave could improve new parents experience and breastfeeding policies well (52, 53). Fathers' fulfillment of their roles and responsibilities has a positive predictive effect on the establishment and growth of children's positive parent-child relationships (54). The international community is also legally requiring and encouraging men to participate in family affairs, maternity care and childcare work, so as to promote the joint commitment of reproductive affairs (54). However, in real life, the phenomenon of a “lack of fatherhood” has become a common problem in society. Therefore, calls for an extension of paternity leave and an improvement fathers' awareness and quality of parenting deserve more extensive attention and support.

Furthermore, the vast majority of working women need to return to work within six months postpartum, which is the main obstacle for them to adhere to breastfeeding (55). In China, women are entitled to 98 days maternity leave, while the expected maternity leave was 233.87 ± 115.87 days, with a median of 180 days (six months) in the study. A study in the USA showed that the length of maternity leave was positively associated with duration of breastfeeding (56). Lactating women were less likely to continue breastfeeding when they return to a full-time job. Also, it has been reported that for each additional month of paid maternity leave, the prevalence of early initiation of breastfeeding increases by seven percentage points, the prevalence of exclusive breastfeeding for six months increases by approximately six percentage points, and the duration of breastfeeding increases by two months. Therefore, it is necessary for China to extend the three periods of leave for pregnancy, maternity, and breastfeeding, add parental leave, while also improving maternity leave and maternity insurance systems.

### 4.6 Low need for breastfeeding promotion and management of breastmilk substitutes

With the ongoing development of Internet, the information about breastfeeding that people have learned is mixed, and some induced consumption has seriously affected their choice of breastfeeding. Our results found that the maternal demand for cultural breastfeeding publicity was significantly lower. In their opinion, they have suffered substantial pressure to breastfeed, particularly in relation to the traditional parenting concepts of elders, physiological factors (such as nipple pain, fatigue, anxiety), and insufficient milk production have added pressure to their breastfeeding. If breastfeeding is promoted again, it will increase feeding anxiety and might be detrimental to mental health (57, 58). However, the vast majority of mothers lack scientific knowledge about breastfeeding, especially primiparas. Therefore, it is necessary to strengthen scientific publicity on breastfeeding to help women and their families fully understand breastfeeding (such as the benefits, difficulties, and methods) in order to help them to make the right feeding decision.

Many studies have pointed out that the promotion and marketing of breast milk substitutes is also a major reason for breastfeeding (59). This study found that women want tighter control of the marketing of breast milk substitutes, but the items “sale of breast milk substitutes for babies under six months in pharmacies” and “infants under six months of age require a pediatrician's permission to add formula” had lower scores. It is not difficult to see that mothers have a high demand for regulating the marking of breast milk substitutes, but they do not want to reduce their own right to choose breast milk substitutes. It is reported that at least 97% of women in China have been exposed to marketing of formula milk before their babies were born (60). In the face of such powerful marketing, the government should pay full attention to managing it. Adhering to the philosophy and practice that the interests of children and parents take precedence over commercial interests, the government should guide society to initiate and maintain breastfeeding.

### 4.7 Influencing factors of breastfeeding guarantee policy need

In traditional Chinese parenting concepts, the nutrient content of human milk becomes lower as the baby's age increases. Furthermore, with the addition of complementary food, parents gradually give up breastfeeding. Many mothers add food supplements too prematurely and think that it is sufficient to breastfeed their baby for 12 months, which our previous qualitative study has also verified. Naturally, their needs and expectations for breastfeeding safeguarding policies are ignored because of them prematurely ignoring breastfeeding.

Mothers' education levels have also been reported to be associated with breastfeeding. Less educated mothers are less likely to choose exclusive breastfeeding or even continue breastfeeding [World Health Organization and the United Nations Children's Fund (UNICEF)] (60). Especially in the most rural areas of China, women have many inappropriate feeding behaviors because of economic and knowledge limitations (23), so they cannot express their demand for breastfeeding guarantee policies. On the other hand, mothers with a higher level of education pay greater attention to the physical and mental health of their children. They will learn about more scientific and better feeding methods through various channels and pay greater attention to the needs of breastfeeding in terms of medical care, amenities, workplace support, and government subsidies (61).

The choice of different payment types by mothers also reflects a family's economic status. Women with higher family economic levels are often more likely to give up breastfeeding because they can afford it (62). They also tend to work and cannot bear the pain and fatigue of breastfeeding. Thus, they pay little attention and have a low demand for a series of policies and measures aimed at ensuring breastfeeding.

Consistent with previous studies, our study showed that the smooth initiation of breastfeeding facilitates the long-term adherence to subsequent breastfeeding recommendations (12). Mothers who choose exclusive breastfeeding will care more and pay greater attention to their baby's diets, also focus on a series of measures that are beneficial to breastfeeding, such as hospital and community support, convenience of breastfeeding in public places, and workplace support (such as flexible working conditions), long maternity leave, government maternity allowance, etc. Also, from promulgating the latest family planning policy (three-child policy) (28), women who want to insist on breastfeeding have more updated expectations and needs for the improvement and implementation of breastfeeding-related guarantee policies.

## 5 Conclusion

This study addressed some gaps in the quantitative research on breastfeeding policies in China and provided more intuitive data and a better understanding of this topic for the Chinese government to promote breastfeeding. In this study, the maternal demand for a breastfeeding guarantee policy is multifaceted and urgent. Maternal education, the baby's age, and current feeding method all have a positive predictive effect on the demand for breastfeeding policies. This also suggests that the government and relevant departments can promote and implement policies based on the relevant influencing factors in the process of improving and implementing policies.

### 5.1 Policy recommendation

Domestic and foreign research on the influencing factors and interventions for individuals, families, and hospitals regarding breastfeeding has been relatively detailed, but there are only a few studies on breastfeeding guarantee policies, especially in China. The formulation and improvement of such policies play a qualitative role in promoting breastfeeding (19). Considering the important leading role of policy, it is very important and necessary to understand the needs of women of childbearing age for breastfeeding guarantee policies. Moreover, according to the specific needs of mothers for relevant policies, the government and relevant departments can implement targeted breastfeeding promotion plans in medical institutions, construct public facilities, as well as ensure workplace support, government support, cultural breastfeeding publicity, and the effective management of breast milk substitutes. In order to further solve the bottleneck problem of persistently low breastfeeding rates, it is important to achieve greater breastfeeding satisfaction. Finally, guiding and controlling the implementation of the breastfeeding promotion plan at the policy level has important practical significance for safeguarding the basic health rights of mothers and infants, improving the quality of life of the population, and promoting coordinated social development, as well as social fairness and justice.

### 5.2 Limitations

Firstly, the convenience sampling and the voluntary nature of the study may lead to some selection bias. The study was conducted in the international city of Shanghai, China, and the sample population was mostly women with higher education and income. Therefore, our findings may not apply to people with lower education or from rural areas. Secondly, some characteristics of the women who refused to participate were not analyzed because of the online data collection method. Due to the limitations of time, manpower, material resources, and other conditions, only one tertiary first-class obstetrics and gynecology hospital in Shanghai was selected for data collection. There may further be some limitations in sample selection and coverage. Thus, the multi-center study will strive to conduct in-depth research in hospitals of different regions and levels. At the same time, the applicability and stability of this survey tool can be further verified. In addition, information on some important factors, such as marital problems and family support, was not collected in our study, which needs to be further confirmed by future studies.

## Data Availability

The original contributions presented in the study are included in the article/Supplementary Material, further inquiries can be directed to the corresponding author.
